# Imaging of dehydration in particulate matter using Raman line-focus microscopy

**DOI:** 10.1038/s41598-019-43959-0

**Published:** 2019-05-17

**Authors:** Peter Ouma Okeyo, Oleksii Ilchenko, Roman Slipets, Peter Emil Larsen, Anja Boisen, Thomas Rades, Jukka Rantanen

**Affiliations:** 1 Department of Pharmacy, University of Copenhagen, Universitetsparken 2, 2100 Copenhagen, Denmark; 2 The Danish National Research Foundation and Villum Foundation’s Center for Intelligent Drug Delivery and Sensing Using Microcontainers and Nanomechanics (IDUN), Department of Health Technology, Technical University of Denmark, Ørsted Plads, 2800 Kgs Lyngby, Denmark; 3 Department of Health Technology, Technical University of Denmark, Ørsted Plads, 2800 Kgs Lyngby, Denmark

**Keywords:** Characterization and analytical techniques, Imaging techniques

## Abstract

Crystalline solids can incorporate water molecules into their crystal lattice causing a dramatic impact on their properties. This explains the increasing interest in understanding the dehydration pathways of these solids. However, the classical thermal analytical techniques cannot spatially resolve the dehydration pathway of organic hydrates at the single particle level. We have developed a new method for imaging the dehydration of organic hydrates using Raman line-focus microscopy during heating of a particle. Based on this approach, we propose a new metastable intermediate of theophylline monohydrate during the three-step dehydration process of this system and further, we visualize the complex nature of the three-step dehydration pathway of nitrofurantoin monohydrate to its stable anhydrous form. A Raman line-focus mapping option was applied for fast simultaneous mapping of differently sized and shaped particles of nitrofurantoin monohydrate, revealing the appearance of multiple solid-state forms and the non-uniformity of this particle system during the complex dehydration process. This method provides an in-depth understanding of phase transformations and can be used to explain practical industrial challenges related to variations in the quality of particulate materials.

## Introduction

Interactions of water with solids are critical for most materials and can have a dramatic impact on their functionality^1^. In the pharmaceutical industry, the majority of the products are marketed as solid dosage forms. Approximately one-third of the existing drug compounds are estimated to have the ability to form hydrates that have different physio-chemical properties in comparison to their anhydrous counterparts^2^. There is an increasing interest in understanding the dehydration pathways of hydrates because of their key importance to processability and storage (preventing unwanted solid-state transformations)^3^. The dehydration of an organic hydrate, when exposed to an external stress such as temperature, pressure or humidity, can result in a mixture of solid-state forms making it challenging to reveal and track metastable intermediates^4^. This has inspired the development of several mechanistic schemes^5^ for the classification of hydrates and most notably among them is the Rouen 96 model by Petit and Coquerel^6^. Rouen 96 assumes that every dehydration process results in the evacuation of water from the crystal structure and the formation of new anhydrous material (NAM) with the optional reorganization of the NAM. Two general classes describe the dehydration pathway and the evolution of NAM in relation to the stable hydrate and the anhydrous product. Class I assumes that the stable anhydrous form has a completely different crystal structure to its stable hydrate, whereas Class II describes the topotactic relationship between the hydrate and the dehydrated product. The Rouen 96 model is based on inorganic materials, that have well-defined pathways of dehydration, but real-life organic material particles contain imperfections resulting in cases, where multiple and crossing pathways can occur simultaneously in the same particle. Morris and Rodriguez-Hornedo also proposed a general structural classification for hydrates due to the location of water in channels or isolated sites in the crystal structure^7^.

Standard solid-state analytical techniques including differential scanning calorimetry (DSC), thermal gravimetric analysis (TGA) and x-ray powder diffraction (XRPD) are commonly used to study the dehydration of organic hydrates^8^. These methods typically require a minimum of a few milligrams of the sample, which means that the analysis is based on several particles and does not consider differences between single particles. In addition, DSC and modulated DSC have a limited sensitivity to observe subtle thermal events, such as the appearance of metastable intermediates or complex overlapping thermal events^9^. Revealing metastable intermediates is of great pharmaceutical importance since they have different physical properties and are known to be more soluble than their stable counterparts^10^. Spectroscopic techniques such as Raman and near-infrared spectroscopy (NIR) are used at different stages of the drug development process and are well suited to monitor hydrate formation and dehydration during processing, but require long measurement times; risking dehydration on the hydrate^11,12^. Computational approaches have recently been used in order to determine the structural changes during dehydration and to identify an amorphous intermediate of ampicillin trihydrate^13^. These approaches provide a molecular-level understanding of dehydration pathways; however, they require experimental methods to confirm.

Herein, we report the use of Raman line-focus microscopy to visualize, at a single particle level, the dehydration pathway of two model drugs; theophylline monohydrate (TP MH) and nitrofurantoin monohydrate (NF MH II). We employ multivariate curve resolution (MCR)^14,15^ and non-negative least squares (NNLS) in the data analysis in order to reveal their metastable solid-state intermediates, and to determine the dehydration pathways of TP MH and NF MH II.

## Results

The solid-state analytical methods applied in this study confirmed the solid-state form of the model compounds of TP to be TP monohydrate (CSD refcode: THEOPH01), TP AH form II (CSD refcode: BAPLOT01) and NF to be NF MH II (CSD refcode: HAXBUD) and NF AH β (CSD refcode: LABJON02) (see Supplementary Figs 1, 2). The thermogram from TGA of TP MH revealed a 9% (one mole of water per mole of drug) weight loss between 55–80 °C. This weight loss occurred as a one-step process when using a heating rate of 10 °C/min. The DSC thermogram of TP MH dehydration resulted in a broad endotherm between 63–95 °C indicative of a channel hydrate^16^. In comparison, the thermogram from TGA analysis of NF MH II revealed a weight loss of 7% (one mole of water per mole of drug) at a heating rate of 10 °C/min. DSC results of NF MH II showed a sharp endotherm between 110–140 °C. The shape of the DSC thermogram from TP MH is suggesting that the dehydration process is a two-step process^17^. Supplementary Figs 3 and 4 show the variable temperature XRPD (VT-XRPD) results for the dehydration of TP MH and NF MH II. The diffractograms of both drugs showed the presence of the intermediate forms, but they were highly overlapped making it challenging to discern the distinct peaks for both drugs. An attempt was made to identify the subtle difference in peaks; however, preferred orientation played a dominating role in these measurements, as both drugs are needle-like in shape. Additionally, the time needed to collect an XRPD pattern is still relatively high and it proved to be a challenge to capture these short-lived solid-state species with the VT-XRPD approach. Our Raman line-focus method circumvents these challenges.

### Measurement setup

**Figure 1:**
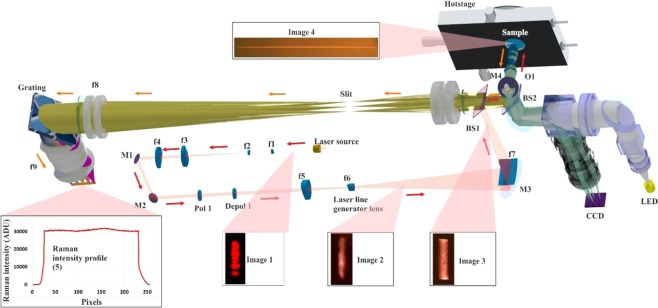
Optical setup of the in-house built Raman line-focus microscope. Where BS – beam splitter, O – microscope objective, M – mirror, f – cylindrical lens, Pol – polariser, Depol – depolariser, LED – light-emitting diode, CCD- visible camera. Image 1 is the laser beam profile from the multimode laser source (785 nm), Image 2 is the laser beam profile after the laser line generator lens (f6), Image 3 is from the laser beam profile before the microscope objective (O1), Image 4 is from the laser beam profile on a given sample (after passing through O1) and the Raman intensity profile (5).

In order to visualise the dehydration pathway of TP MH and NF MH II during heating an in-house built Raman line-focus microscope (Fig. 1) was used (see “Methods section”). Temperature and time-dependent Raman measurements were performed in combination with a Linkam hot stage (FTIR600 stage, T95 Linksys controller) in order to image the single particle of the drug during heating. A single particle was fixed with an aluminium clamp to ensure that the particle was immobile during the measurements. The 1.8 mm laser line was illuminated on the particle during collection of the Raman spectra yielding 220 spatially separated spectra per measurement. The optical images of the crystal(s) were collected simultaneously (Supplementary Figs 5, 6). One of the main benefits of using hydrates in these experiments is due to the physical change in the particles from transparent to opaque as a result of dehydration. This is due to the formation of nucleation events that are occurring on the surface of the particle resulting in the formation of small-sized crystallites that can be seen visually using optical microscopy by the formation of dark regions^18^. The measured multiple single particles of NF were recrystallized from NF AH β spiked with multiple single particles (different shapes and sizes) of NF MH I, NF AH α and NF AH β. A depolariser was used in order to eliminate particle orientation as a contributing factor to all the experiments.

### Experimental solid-state characterisation of TP and NF

It is challenging to obtain pure Raman spectra of the metastable intermediates as they overlap with their stable counterparts. It is equally challenging to capture these short-lived species with diffraction-based methods as shown by our VT-XRPD results and typically, synchrotron-based radiation is needed to observe these intermediates^19^. Therefore, in order to confirm the presence of the metastable intermediates of TP and NF, multiple isothermal experiments between 45–130 °C 90 minutes (each experiment) were conducted using only a single particle per experiment and the residual intensity maps were calculated from the raw Raman spectra taking into account the stable forms^20,21^. This was a critical step in the experiments because by subtracting the stable hydrate and anhydrous responses from the hyperspectral Raman data we were able to study the dynamics of only the metastable intermediates of TP and NF. This is a method that has previously been applied to studying multicomponent systems (see refs^16,17^).

**Figure 2:**
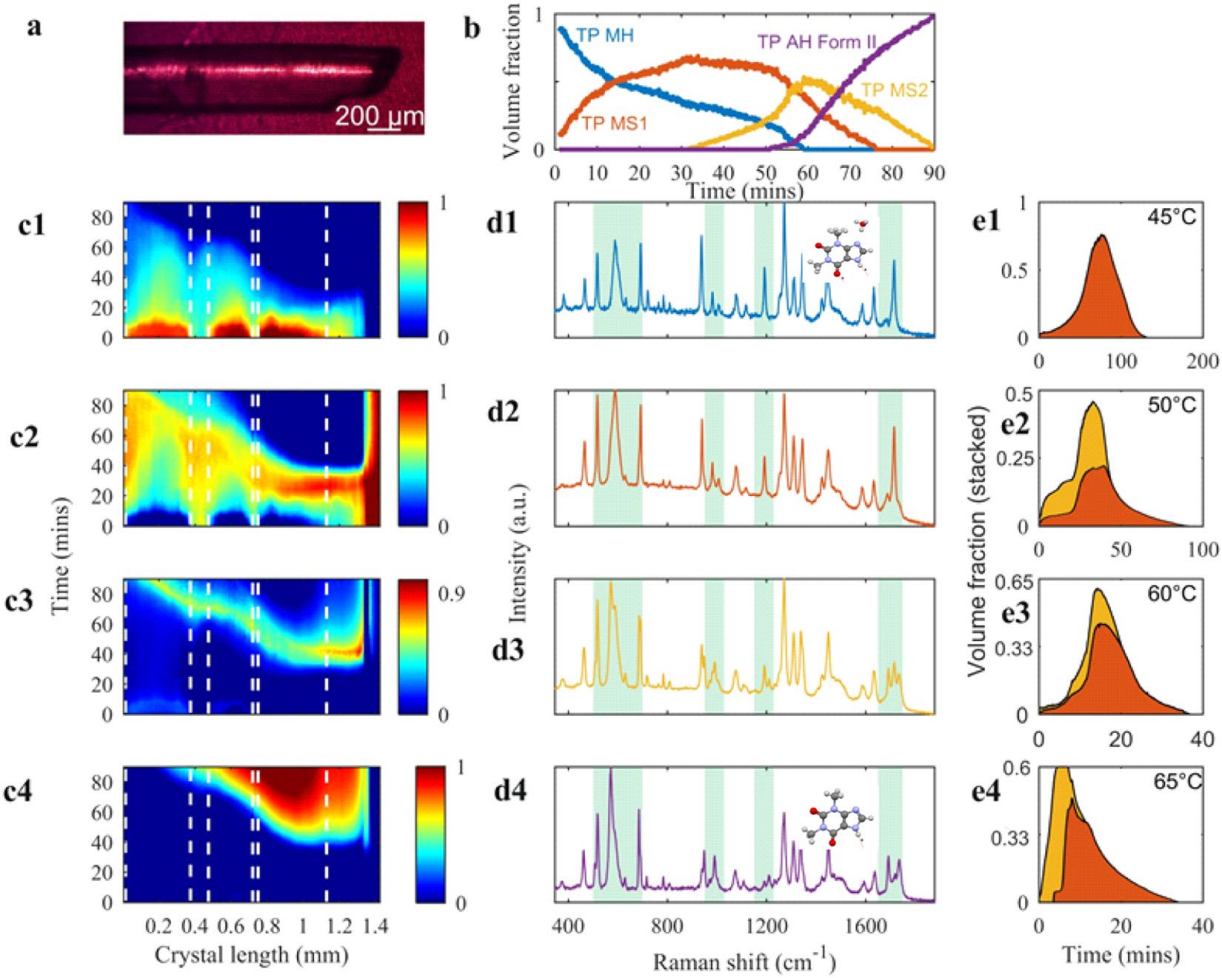
MCR and NNLS decomposed results of hydrate and anhydrous solid-state forms of theophylline. (**a**) Optical image of TP MH at 25 °C with laser line illumination (white line) from the Raman microscope (**b**) concentration profile of solid-state forms during isothermal dehydration at 50 °C for 90 minutes starting from TP MH to TP AH form II, via TP MS1 and TP MS2 (**c1–4)** chemical concentration maps (cut out artefacts from 1.8 mm laser line) of TP MH **(c1)**, TP MS1 **(c2)**, TP MS2 **(c3)**, TP AH form II **(c4)** in the particle during dehydration where the laser line is illuminated, (**d1–4**) Raman spectra for TP MH, TP MS1, TP MS2 and TP AH form II respectively, (**e1–e4**) area plots showing only the dehydration profiles of TP metastable intermediates at four isothermal conditions (45, 50, 60 and 65 °C) where the colour under the area plots matches the TP metastable intermediates as shown in the concentration profiles. The stable forms of TP were taken into consideration for the area plots.

In order to understand the dynamics of the metastable intermediates of the model particles, concentration profiles and maps were obtained from the Raman data using MCR and NNLS. The isothermal dehydration of a single particle of TP MH was conducted from 45–65 °C for 90 minutes (each experiment) using the Raman line-focus setup and the hot-stage (Fig. 2a). These experiments showed the complex dehydration pathway of TP MH and they revealed a new metastable intermediate (TP MS2). The concentration profiles at 50 °C for 90 minutes obtained after MCR analysis of the hyperspectral Raman data showed an initial decrease in the concentration of TP MH accompanied by a simultaneous increase in the concentration of TP MS1 (Fig. 2b). The decrease of TP MS1 is followed by an increase in the concentration of TP MS2 via two steps after 32 min of the dehydration process at 50 °C. TP AH form II increases in concentration up to the end of the experiment in a mixture with TP MS2. Reported literature has shown that the dehydration pathway of TP MH to TP AH form II includes only one metastable intermediate, which we refer to as TP MS1; however, to the best of our knowledge, we are the first to experimentally reveal TP MS2. The concentration profiles were able to highlight the overlap of the solid-state forms during dehydration, but do not give information with regard to the spatial location of both intermediates in the TP particle; hence, chemical concentration maps were needed.

In order to visualise the heterogeneous distribution of the four solid-state forms of TP from its single particle, NNLS was applied on the Raman data to obtain four chemical concentration maps of TP (Fig. 2c1–4). The TP MH map showed specific regions in the single particle (0.02–0.38 mm, 0.48–0.72 mm, and 0.75–1.13 mm) with high concentrations of TP MH and this is due to local defects that were present in the particle. TP MH and TP MS1 appear at the same time as in the concentration profile but are located spatially in different parts of the particle. This level of spatially resolved information cannot be obtained with DSC and TGA, as they require bulk samples for analysis. Fig. 2c1–2 show that TP MH and TP MS1 overlap and diffuse throughout the particle at the end of the experiment at the ‘defect-rich’ areas. After 30 minutes, a gradual decrease in the concentration of TP MS1 as it spreads throughout the particle. For 45 minutes, TP MS1 and TP MS2 were overlapping in different concentration profiles. This could be linked to their structural similarity as shown by the recorded Raman spectra and potentially a small energy barrier that exists between these two metastable forms. The concentration profile and map show that TP AH form II appears after 50 minutes when both metastable intermediates of TP were present in varying concentrations.

Raman spectra of the four different solid-state forms of TP were obtained during previously described experiments from the MCR analysis. The spectral changes during dehydration are shown in Fig. 2d1–4. A comparison between TP MH and TP AH form II showed two peaks (1690 and 1730 cm^−1^), indicative of TP AH form II and are due to C=O, C=N and C=C stretching modes^22^. This is indicative of the loss of hydrogen bonding^23^ related to the water molecules present in TP MH. As shown in Fig. 2d2–3, the spectral regions 500–750 cm^−1^ (C–C aliphatic chain vibrations) and 1650–1750 cm^−1^ are where the most differences were noted between the spectra of the four forms. The Raman spectra of TP MS1 and TP MS2 differed in the regions around 1150 cm^−1^, 1230 cm^−1^, and 1750 cm^−1^ due to deformation of the imidazole and pyrimidine ring. This deformation is critical in revealing the small structural differences between the metastable intermediates even though they are overlapping during dehydration^24^. The crystal structures of TP MH and TP AH form II are published^25^. To the best of our knowledge, we are the first to publish the mid-frequency Raman spectra of TP MS1 and TP MS2. However, their structures have not been determined due to their metastable nature.

The isothermal experiments (45, 50, 60, and 65 °C) demonstrated the solid-state transformation between TP MS1 and TPMS2 (Fig. 2e1–4), where it should be noted that the result in the Fig. 2e2 is from another TP MH particle than the result presented in the Fig. 2b. The stable forms were taken into account, however, in the Fig. 2e1–4 only the metastable intermediates have been plotted. Both metastable intermediates are highly overlapped in the isothermal conditions 50–65 °C. At 45 °C, only TP MS1 was observed, but at 50, 60 and 65 °C both TP MS1 and TP MS2 were observed. The appearance of TP MS1 and TPMS2 is slower at 45 and 50 °C and faster at a 60 and 65 °C. At each isothermal condition, the dehydration profiles of the metastable forms are different with only TP MS1 appearing at 45 °C, but TP MS2 appearing at 50, 60 and 65 °C. TP MS2 appears at the same time as TP MS1 at 60 °C and before TP MS 1 at 65 °C.

**Figure 3:**
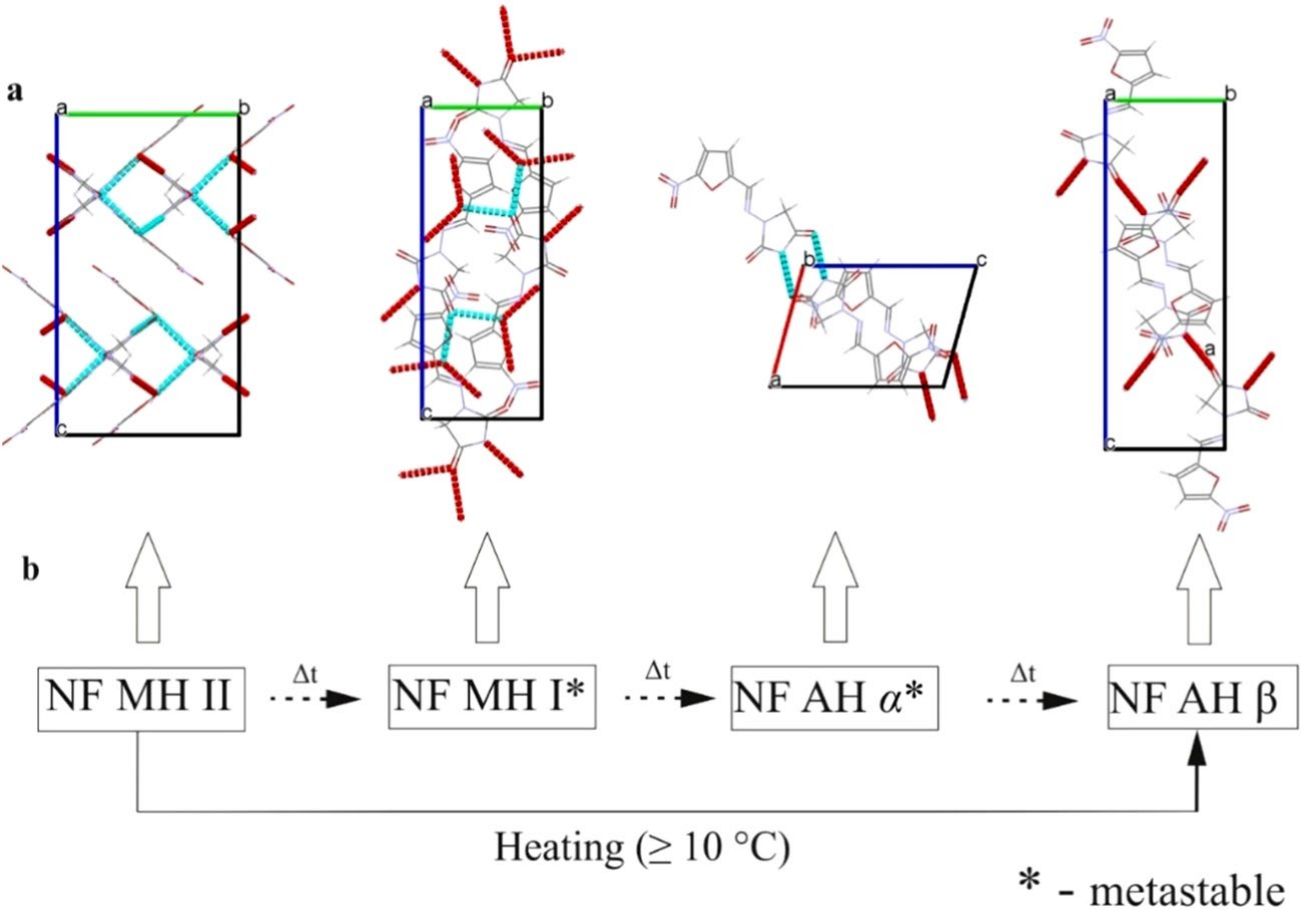
Solid-state forms of NF (**a)** 3-D representation of NF MH II, NF MH I, NF AH α and NF AH β respectively (light blue lines indicate hydrogen bonding) taken from the CSD (**b**) proposed complex dehydration pathway of NF MH II to NF AH β, where Δt is the change in time at a given temperature.

Since our Raman based method was able to reveal a new metastable form of TP, a more complex model compound (NF) was studied where a limited amount of work has been done on its dehydration pathway. The crystal structures of the four solid-state forms of NF have been published^26^ and formed the foundation for our proposed dehydration pathway for NF MH II to NF AH β. The crystal structures presented in Fig. 3a were taken from the Cambridge Structural Database (CSD) in order to show the differences in the arrangement of the water molecules in each form of NF and how this translates into a complex dehydration pathway due to the presence of two metastable intermediates (NF MH I and NF AH α). Hydrogen bonds play an important role in the stability of hydrates. It has been reported^27^ that the C=O, N–H and C–H groups are strongly affected by hydrate formation and it is thought that the dehydration of a hydrate also strongly affects these vibrational modes via intermolecular bonding. Figure 3b shows the proposed dehydration pathway based on the hydrogen bonding arrangement from NF MH II to NF AH β. NF MH II consists of a complex hydrogen bonding herringbone arrangement (zig-zag) whereas NF MH I has a chair like the arrangement of the hydrogen bonds indicating a partial loss of water. The anhydrous forms possess a planar conformation with dimers forming a head-to-head bond between NF molecules with NF AH α having two N-H···O bonding patterns whereas β has one.

**Figure 4:**
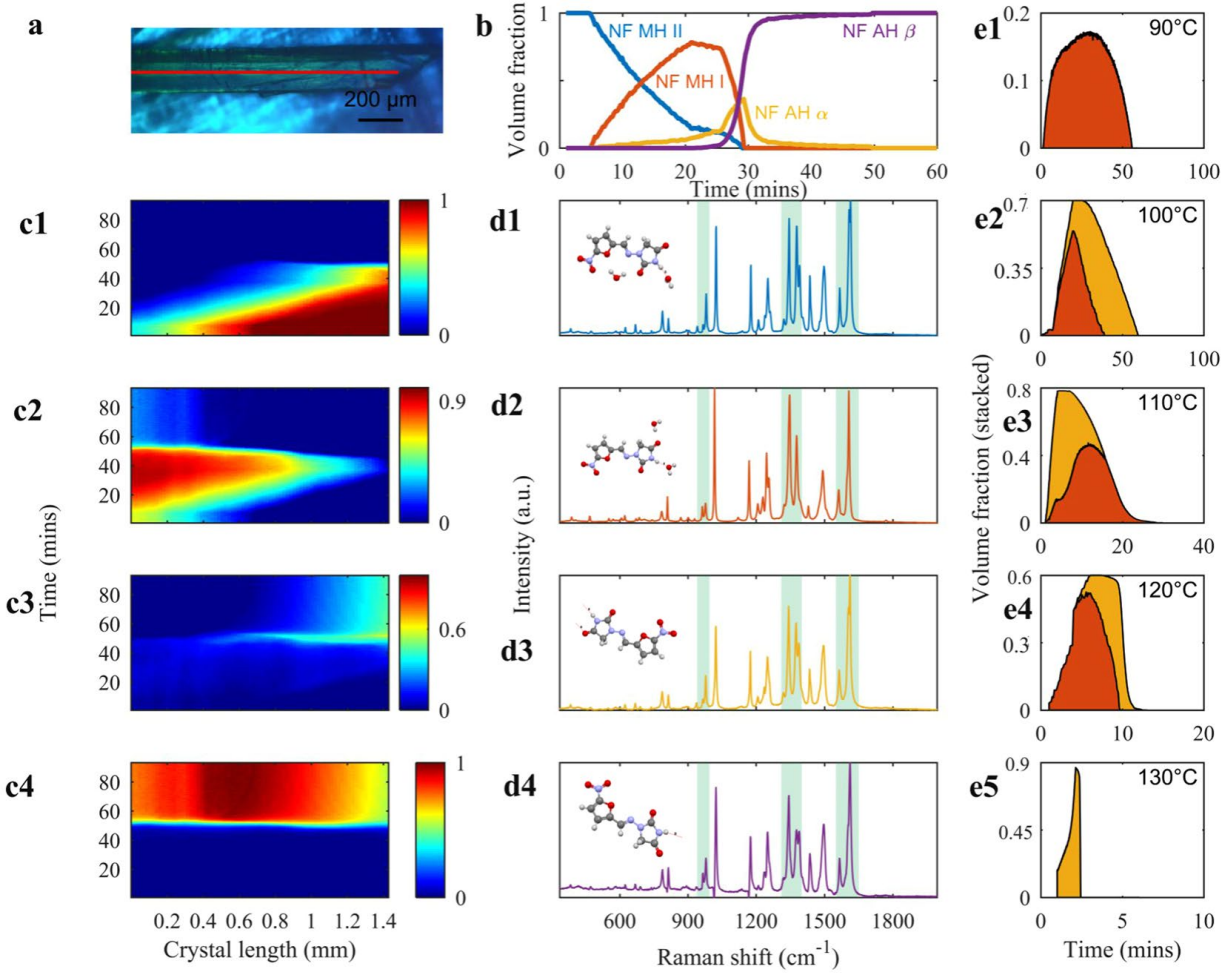
MCR and NNLS decomposed results of nitrofurantoin solid-state forms. (**a**) Optical image of NF MH II at 25 °C from the Raman microscope. (**b)** concentration profiles of solid-state forms during isothermal dehydration at 120 °C for 90 minutes from NF MH II to NF AH β via, NF MH I and NF AH α (**c1–4**) chemical concentration maps (cut out artefacts from 1.8 mm laser line) of NF MH II **(c1)** NF AH β **(c2)** NF MH I **(c3)** NF AH α **(c4)** in the particle during dehydration where the laser line is illuminated (**d1–4)** Raman spectra of NF MH II, NF MH I, NF AH α, NF AH β. (**e1–4)** area plots showing only the dehydration of NF MH I and NF AH α five isothermal conditions (90, 100, 110,120 and 130 °C) where the colour under the area plots matches the NF metastable intermediates as shown in the concentration profiles. The stable forms of NF were taken into consideration for the area plots.

Based on the crystal structure understanding of the NF forms, isothermal dehydration of a single particle of NF MH II was conducted in the same manner as for TP MH, but at isothermal conditions from 90–130 °C each for 90 minutes using the in-house Raman line-focus setup and a hot-stage (Fig. 4a, Supplementary video 1). MCR and NNLS analysis described in the methods section was applied to the hyperspectral Raman data to reveal the concentration and spectral profiles of NF forms during the isothermal experiments. Figure 4b shows that at 120 °C from 5 minutes, both hydrates of NF were overlapping, but NF MH II is decreasing in concentration and NF MH I increasing, confirming a solid-state transformation of one monohydrate into another structurally different monohydrate. NF AH α also appeared at the 10-minute mark with both monohydrates. These two metastable intermediates appeared simultaneously as seen with TP during isothermal dehydration. The decrease in the concentration of NF AH α at 30 minutes coincides with the formation of NF AH β at the same time point and increases until NF AH β reaches a plateau. The four chemical concentration maps of NF show that the dehydration is occurring from the centre of the particle to the boundaries heterogeneously with a gradual decrease in the concentration of NF MH II from the middle of the particle to its edge^28^ (Fig. 4c1–4). In contrast to the dehydration of TP MH to that of NF MH II occurs at a higher temperature (≥90 °C), which is unusual and this could be because it is an isolated site nature of this hydrate. The results showed that NF MH I is present at higher concentrations inside the particle whereas residual amounts of NF AH α are present inside the particle at the beginning of the experiment and it protrudes to the edge of the particle where a higher concentration is present. The concentration profiles of NF metastable intermediates are different and this could be linked to their crystal structure as well as NF MH I being trapped in specific local regions in the crystal. NF AH β appears during the disappearance of the hydrated counterparts and is present from the centre to the end of the NF particle. The Raman spectra highlight the structural difference of the NF forms.

The four Raman spectra of NF solid-state forms were obtained from MCR analysis. In Fig. 4d1–4 they have pronounced differences that can be attributed to their conformational arrangement^29^. NF MH II and NF MH I differ in their C=N linkage between the nitrofuran and the hydantoin moieties (1615 cm^−1^). The regions in the Raman spectra of NF AH α and NF AH β that have the most pronounced differences were 950–1000 cm^−1^, 1200–1300 cm^−1^, 1310–1400 cm^−1^, 1550–1650 cm^−1^.

The isothermal experiments (90, 100, 110, 120, and 130 °C for 90 minutes) demonstrated the solid-state transformation between two monohydrates (hydrate-hydrate transformation from NF MH II into NF MH I) between 90–120 °C (Fig. 4e1–5) where it should be noted, as earlier, that the Fig. 4b,e4 are from different crystals of NF MH II. The isothermal experiments of NF showed that dehydration occurred at a slower rate at below 110 °C in comparison to 120 and 130 °C. In addition, between 100 °C and 120 °C, both the NF MH I and NF AH α are present, whereas only NF AH α at 130 °C for a few minutes. The difference in the dehydration rates of both 120 °C isothermal runs is due to a difference in particle size. As with TP, the dehydration profiles of the metastable forms are different at each isothermal condition.

### Visualization of the solid-state form diversity in real particles

**Figure 5:**
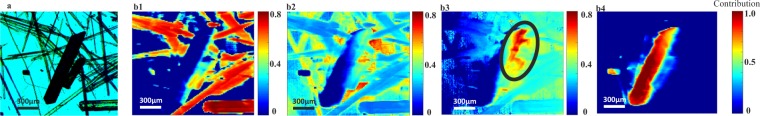
Multiple particles of NF solid-state forms analysis using the Raman line-focus method at room temperature. (**a**) Optical image of multiple particles of NF. (**b1**) NF MH II. (**b2**) NF MH I (**b3**) NF AH α and (**b4**) NF AH β obtained after NNLS of a depolarised Raman map.

The results presented above capture the dehydration of TP MH and NF MH II using only a single particle of both compounds. However, in order to have a more direct comparison of the Raman line-focus method with the standard thermal analytical methods such as DSC and TGA, a Raman line-focus mapping option in an area of 1170 × 2109 μm with a step size of 8 μm was performed using a real-life powder sample with different particle sizes and shapes (Supplementary Fig. 7). A batch of NF particles (Fig. 5a) that contained a mixture of its solid forms was mapped in order to simulate potential real-life temperature-driven phase transformations (Supplementary video 3–5). The maps obtained from NNLS (Fig. 5b1–4) were cut to show specific regions of interest. The maps confirmed the presence of four different solid forms of NF in the randomly orientated particles as was identified in the single particle experiment for NF. Figure 5b3 shows that even though the single particle of NF (circled) appears to be fully dehydrated based on the visual appearance of the optical image (dark particle) there are residual amounts of NF AH α present within the particle.

Previous literature has shown the particle size dependence of the dehydration process^30^, which motivated us to explore the possibility to visualize multiple particles at the same time. Typically, DSC and TGA analysis will take an average thermal signal resulting from all the particles during the heating, making it challenging to reveal subtle thermal events and overlapping signals that could be taking place in these particles, such as the appearance of metastable intermediates^31^. However, the Raman line-focus method was clearly able to identify NF MH I and NF AH α in multiple single particles using NNLS of a depolarised Raman map.

## Discussion

The experiments show the capability of our approach to visualize and spatially locate the different solid-state forms of TP and NF at a single particle level. DSC and TGA show the quantitative amount of water loss for hydrates, however, they are not able to spatially resolve the complex dehydration pathway at a single particle level. The concentration profiles and the chemical maps of the solid-state forms provided a deeper insight into the dehydration pathway of both model compounds, in particular, the dynamics of the metastable intermediates. Both model compounds were structurally very different resulting in different dehydration pathways. TP MH consists of a channel type crystal structure^32^, where water formed directional bonding between the water (anisotropic dehydration) and TP molecules, whereas water of crystallization in NF MH II is located at isolated sites with van der Waals forces and hydrogen bonding resulting in planar conformation with direct water-to-host bonding. The dehydration process of NF MH II can be considered ‘destructive’ as it contains two hydrogen bond donors and seven hydrogen bond acceptors, which could^33^ play a role in the NF MH II transformation to NF MH I at high dehydration temperatures. The single particles used in these experiments contained defects and this contributed to their heterogeneous dehydration, with water disappearing at different rates in specific regions of the particle^34^. Taking into account the Rouen 96 model (largely based on observations with inorganic materials), our results show that there are crossing pathways of water release from TP MH and NF MH II. This is because these model compounds represent real-life particles, which are inherently complex^35^.

Currently, there are four known forms of TP, and form IV is reported as the thermodynamically stable form at room temperature^36^. Seton *et al*. also noted the important role of the dimers between the TP and water molecules ensuring the physical stability of the monohydrate network. TP MH is known to convert to its anhydrous form II and this is the form that was found at the final stages in our experiments^37^. The reported appearance of TP MS1^38,39^ show that it can transform to its stable anhydrous form during storage over a period of days or months depending on the environmental conditions^40^, hence TP MS1 was present at the beginning of the isothermal measurements. To the best of our knowledge, we are the first to record experimentally the Raman spectra of TP MS1 and TP MS2 in the region 400–1800 cm^−1^; however, computationally assisted approaches could give additional insight into these complex dehydration pathways^41,42^. As previously noted, it was surprising for TP MS1 and TP MS2 to be present in the experiment for over an hour. This could be because we have created experimental conditions in which the metastable form is stable and therefore appears for several minutes rather than seconds^43^.

For NF MH II dehydration, previously reported literature using standard analytical techniques indicates that the dehydration pathway of NF MH II to NF AH β (120–145 °C) is a one-step process^44^. However, we discovered using our method that it is a complex three-step process due to the presence of two known metastable intermediates. The proposed dehydration pathway of NF MH II to NF AH β is based on the crystal structures that highlight the hydrogen bonding arrangement, which plays an important role during dehydration. Although there are pronounced differences between these forms, the metastable intermediates are still overlapping with the stable counterparts indicating that the energy difference could also be small between the four forms. This observation follows the Ostwald’s rule of stages that states that matter at a high-energy state does not necessarily transform directly into its most thermodynamically stable form but can pass through several metastable intermediates^45^.

Measuring multiple particles can be useful in resolving the particle-size dependence of metastable intermediates. This approach provides the possibility to resolve essential information regarding the transformation from hydrated to anhydrous forms in particulate materials, and the spatial location of metastable intermediates^46,47^. The crossing pathways of water release from particulate matter can have a detrimental impact on the physical stability of the drug and potentially its bioavailability. The limitations of our approach are that fluorescence can be a challenge with the Raman analysis. The Raman line-focus method can be used in a variety of different fields of research that require analysis of phase transformations at the particulate level and would naturally complement molecular level investigation of phase changes using a computational method such as molecular dynamics^48^. Mechanistic models and computer simulations are still challenging to implement for real-life particulate systems. Our findings are pointing towards mechanistic models based on combining information at different levels: spectroscopic imaging visualizing molecular level phenomena, such as solid-state diversity during dehydration in a single particle, combined with optical microscopy to visualize particle level phenomena, such as crack density related to this phase transformation. This can provide a basis for multiscale mechanistic models^49^ and experimental validation of computer simulations. This approach would have similarities with the deep learning process in cancer diagnosis based on imaging of cellular samples^50^.

## Conclusion

Here we demonstrated experimentally that Raman line-focus microscopy can be used to investigate different crystalline solid-state transformations and reveal metastable intermediates that are of pharmaceutical relevance at the single particle level. The obtained concentration profiles and maps with the usage of MCR and NNLS analysis showed that the dehydration pathways of TP MH and NF MH II went through two intermediates and this approach was able to spatially locate them, which classical thermal techniques cannot do. This resulted in the discovery of TP MS2 and the dehydration pathway of NF MH II to its NF AH β. The dehydration pathway of NF MH II leads to a hydrate-hydrate transformation of one monohydrate structure into another structurally different monohydrate at dehydration temperatures. In addition, a Raman line-focus mapping option was used to analyse real-life particles, highlighting the diversity of solid-state forms present during dehydration. It is envisioned that this method can be used in providing an in-depth understanding of phase transformations, as well for explaining the practical industrial challenges related to variation in the quality of particulate materials.

## Methods

### Materials and general preparative methods

All particles were stored at room temperature before conducting thermal measurements with the Raman microscope. A single particle of theophylline monohydrate (Cambridge Structural Database ref code: THEOPH01)^51^ and nitrofurantoin monohydrate (CSD refcode: HAXBUD)^52^ was used for these experiments. Particles from the same batch were used in order to carry out the Raman measurements.

50 g of anhydrous TP (Sigma Aldrich) was dissolved in distilled water (1 L, 80 °C) and needle-like single particle (500 μm–3 mm) of TP MH appeared within 3–5 days from slow evaporation crystallization. Bulk powder of nitrofurantoin (obtained from Unikem A/S, Copenhagen, Denmark) was identified as the stable anhydrous form (β) using XRPD. NF MH II was recrystallized from one gram of bulk powder nitrofurantoin (Unikem A/S, Copenhagen, Denmark) from a hot (55 °C) acetone-water solution (3:1). The solution was cooled to room temperature and within 3 days yellow needle-like particles (500 μm–2 mm) formed. NF monohydrate (NF MH I) was recrystallized from 75 mg of NF (Unikem A/S, Copenhagen, Denmark) dissolved in 30 cm^3^ of a hot (55 °C) acetone: water mixture (2:1). The solution was then left in a sealed vessel and maintained at 40 °C allowing for slow evaporation. Plate-like particles (100 μm–2 mm) appeared within 4 days. An optical microscope (LEICA DM LM) was used to identify a single particle by observing the clarity of the particle and whether a large number of crystal defects were present.

### Sample characterization

Particles of TP MH and NF MH II were characterised by polarised light microscopy, x-ray powder diffraction (XRPD), differential scanning calorimeter (DSC) and thermogravimetric analysis (TGA) and Raman microscopy.

### X-ray powder diffraction (XRPD)

A PANalaytical X’pert PRO X-Ray Diffractometer (purchased from PANanalytical B.V., Almelo, Netherlands) consisting of a θ/θ goniometer and a solid state PIXcel detector was used for solid-state form identification and verification. The radiation was nickel-filtered CuK_α_ (λ = 1.5418 Å) generated at a tube voltage of (45 kV) and current (40 mA), respectively. The samples were scanned in reflection mode between 5° and 35° with a scan speed of 0.06734° 2θ and a step size of 0.0263° 2θ. The data were analysed using the X’Pert Data Collector software (PANalytical, Almelo, Netherlands). The measurements were done in triplicate. The variable temperature-XRPD measurements were all performed using a steel sample holder (0.2 mm in depth) on an Anton Paar CHC chamber (Anton Paar GmbH, Graz, Austria). The temperature was controlled using a TCU 110 Anton Paar GmbH controller. A scan speed of 0.328 was used for NF and TP temperature measurements. For NF MH II the measurement was performed at room temperature then the temperature was ramped to 120 °C at 35 °C/min and held for 120 mins with an exposure time of 10 mins. For TP MH, the temperature was raised to 50 °C at the same heating rate and held for 90 minutes. The particle size that was used for the temperature measurements were between 50–150 μm.

### Thermal analysis

A Discovery DSC (TA Instruments-Waters LLC, New Castle, USA) was used to perform the DSC measurements. The instrument is controlled by TRIOS software (TA Instruments, New Castle, DE, USA). Samples around 7 mg were placed into T-zero aluminium pans and sealed. Samples were subjected to a controlled heating rate of 10 °C/min under nitrogen purge (40 mL/min). The measurements were done in triplicate.

The water content in TP MH and NF MH II was determined using a Discovery TGA (TA Instruments, New Castle, DE, USA), which was controlled by TRIOS software (TA Instruments, New Castle, DE, USA). Samples around 7 mg were analysed in an aluminium pan and heated at 10 °C/min from 25 °C to 100 °C for TP MH and 25 °C to 200 °C for NF MH II. The measurements were done in triplicate.

### Visualisation of crystal structures

The CSD was used in order to visualize and understand the intermolecular arrangement of the bonds, unit cell dimensions, and faces of the crystals of the different TP forms (CSD ref code: THEOPH01, BAPLOT01^53^ and NF forms (CSD refcode HAXBUD, HAXBUD01,LABJON01^54^, LABJON02) using Mercury 3.8 software that is provided by the Cambridge Crystallographic Data Centre, UK.

### Raman line-focus microscope setup

An in-house built Raman microscope that is based on a line-focus method was used to track the structural changes that occurred in the single particle during dehydration. The setup consisted of three main units: laser delivery optical system, a visible light microscope and a Raman scattering optical system. A high power multimode laser (0.5 W, 785 nm) is used to generate an inhomogeneous laser line, which propagates through a number of cylindrical lenses in order to achieve a homogenous beam on the particle (s). The 1.8 mm in length laser line was focused on the particle fixed on a Linkam FTIR600 hot stage during heating at a controlled rate at 40–55% relative humidity (RH). The alignment of the particle onto the laser line was performed manually in two stages: firstly, by placing the particle within the visual field of the microscope and secondly, by obtaining a more precise alignment using the x, y and z stage controlled by the in-house Raman data acquisition software. An exposure time 8 seconds for the acquisition of 220 Raman spectra along the line length was used for all the isothermal measurements in the main body of the paper. The laser intensity at 785 nm was 100 μW/μm for all experiments. The line-focus method enables measurements that are two orders of magnitude faster than traditional point illumination Raman microscopy and requires less power per point^55^. At 100 mW power using a point measurement mode, this would generate 33.3 mW/μm^2^ whereas with laser line illumination this would generate 0.15 mW/μm^2^. This is a significantly less power point thereby reducing the likelihood of damage to the particle due to the laser.

Wavelength and spectrum-dependent intensity calibration of the Raman system were performed using toluene and cyclohexane. The intensity calibration included corrections on the quantum efficiency of CCD and the transparency of optical elements in the system. The wavelength calibration was done automatically during the start of the system software, and spectrum-dependent intensity calibration is done periodically every month. The Raman spectra were collected according to the international standard guides ASTM 1840, ASTM E2911.

### Raman optical system

A series of images (1–4) of the different laser beam profiles were captured in order to illustrate how a homogeneous laser line was achieved with the use of cylindrical lenses (f1–f7). Image 1 consists of five different spatial modes in the vertical direction as generated from the laser source. These five spatial modes pass through f1 to f5 resulting in image 2, which shows a laser beam profile that is not yet homogenous. It is only after image 3 goes through f7 that a homogeneous laser line in the vertical direction is achieved. Image 4 is based on the field of view (FOV) of the microscope objective (10× NIR objective with a FOV of 2.2 mm). The laser beam is then reflected from the dichroic beam splitter (BS1) before it reaches the microscope objective (O1) then passes through a set of mirrors to O1. BS2 is used to combine the laser and visible light beams thereby allowing for real-time visual inspection and collection of the Raman spectra. The light projected from the light-emitting diode (LED) is cut off from the Near Infrared spectrum of the Raman shift (785–1000 nm) using an edge filter (Ed3). The visual inspection of the crystal (s) during dehydration was very useful for the monitoring of the crystal (s) opacity, especially in “defect-rich” areas.

The microscope was designed in a manner that allowed the sample to be scanned in the X, Y and Z directions without being moved. This was important in order to ensure no misalignment of the laser and visible light beams. In order for no misalignment to take place during measurements BS2, Mirror 4 (M4) and O1 moved as one unit in the x-direction and M4 and O1 moved as one unit in the y-direction. As previously stated, image 4 is based on the projection of the laser line from the microscope objective. The Raman spectra were collected on the particle and simultaneously collected on a deep-cooling spectroscopic charged coupled detector (CCD). This setup provided the unique capabilities for simultaneous Raman mapping on an entire particle during temperature ramping or under isothermal conditions. The spectrograph is protected under a patent (DTU Patent Application No. PA201870044) and its aberration corrected imaging design provides Raman spectra from 350–2300 cm^−1^ at 785 nm.

### Multivariate curve resolution (MCR) and non-negative least squares (NNLS) methodology

MCR-ALS^56,57^ and NNLS were both performed in MATLAB R2017a. Raman experiments resulted in a matrix that has two dimensions [t, S(ν)], where t corresponds to the temperature or time points, while S(ν) corresponds to Raman spectra. We registered 220 spectra from the laser line at each temperature/time point, and all spectra were grouped as follows: M_line_ = [t_1_(S(ν)_1_, S(ν)_2_, … S(ν)_220_), t_2_(S(ν)_1_, S(ν)_2_, … S(ν) _220_), … t_n_(S(ν)_1_, S(ν)_2_, … S(ν) _220_)], where n is the number of temperature/time points. In order to extract information about the concentration and spectral profiles of the studied model compounds, we used only one lateral point on the laser line that had the highest Raman spectrum signal to noise ratio (SNR).

When using MCR-ALS the following constrains were applied: non-negativity on spectral and concentration profiles, closure and unimodality constrains on concentration profiles. The “mcr_main” toolbox was downloaded from the “Multivariate Curve Resolution homepage”. Before running the “mcr_main” toolbox a spectral library for the stable hydrate and anhydrous form for TP and NF dehydration experiments were created with four rows of which two-reference spectral matrix were filled containing the Raman spectra of the stable forms of the model compounds. These stable forms were used as the spectral profile constrains. The two unfilled rows in the library spectral matrix were defined as not a number (NaN). After running the “mcr_main” function on MATLAB, the rows that filled the two NaN rows contained the metastable intermediates. The best lack of fit (LoF) was reached in the case of using four Principal Components (PCs) for TP analysis (two metastable intermediates observed) and four PCs for NF analysis (two metastable intermediates observed). The averaged matrix of residuals was found to be less than 0.1% after the iteration process. As a result, we obtained four concentration and spectral profiles from our dataset from the dehydration of TP MH and NF MH II. The obtained spectral profiles of metastable intermediates of the model compounds were used as a spectral library for NNLS analysis of line-focus Raman matrixes M_line_. NNLS results are shown in Fig. 2b–d4 and Fig. 4b–d4.

## Supplementary information

supplementary information

video 1

video 2

video 3

video 4

video 5
