# Supplementary material for: Imaging of dehydration in particulate matter using Raman line-focus microscopy
Source: Sci Rep. 2019 May 17;9:7525. doi: 10.1038/s41598-019-43959-0 (PMC6525166; doi:10.1038/s41598-019-43959-0)
Supplement: Supplementary file 1 — supplementary information [file 41598_2019_43959_MOESM1_ESM.pdf]

Supplementary Information

Imaging of dehydration in particulate matter using Raman line-focus microscopy

Peter Ouma Okeyo[1,2,3], Oleksii IIchenko[2,3], Roman Slipets[2,3], Peter Emil Larsen[2,3], Anja Boisen[2,3], Thomas Rades[1], Jukka Rantanen[1]

[1]Department of pharmacy, University of Copenhagen, Universitetsparken, 2, 2100, Copenhagen, Denmark

[2]The Danish National Research Foundation and Villum Foundation's Center for Intelligent Drug Delivery and Sensing Using Microcontainers and Nanomechanics (IDUN), Department of Health Technology, Ørsted Plads, 2800 Kgs, Lyngby, Technical University of Denmark
[3] Department of Health Technology, Technical University of Denmark, Ørsted Plads, 2800, Kgs. Lyngby, Denmark

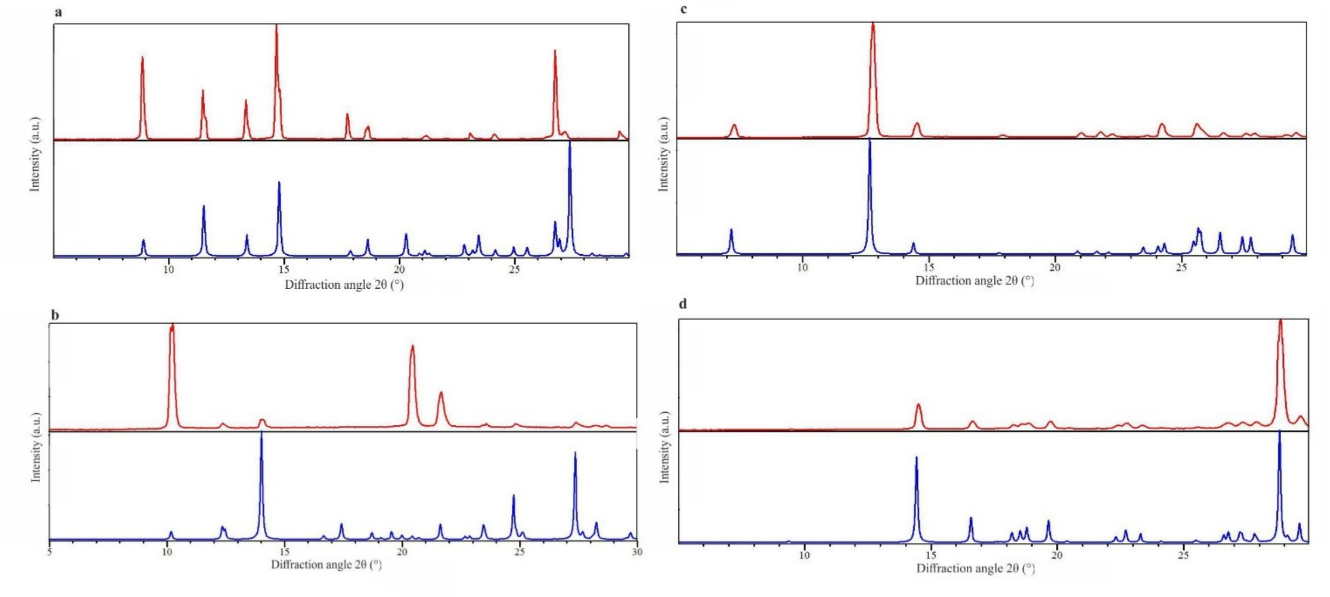

Supplementary figure 1. Experimental (in red) simulated (in blue) XRPD diffractograms. a) The diffractograms of TP MH, THEOPH01 **b)** TP AH form II, BAPLOT and **c)** NF MH II, HAXBUD **d)** NF AH β, LABJON02.

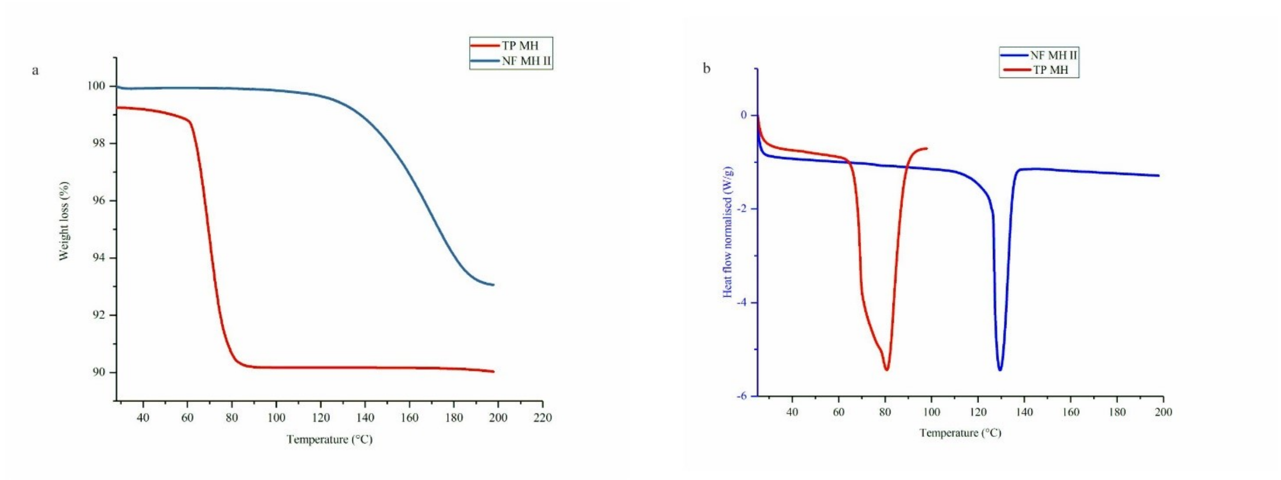

Supplementary figure 2. a) TGA thermograms, and b) the DSC thermogram of NF MH II (blue) and TP MH (red) at a heating rate of 10 °C/min. NF MH II showed a sharp endotherm between 110-140 °C and TP MH thermogram showed a broad, two-step endotherm between 63- 95 °C.

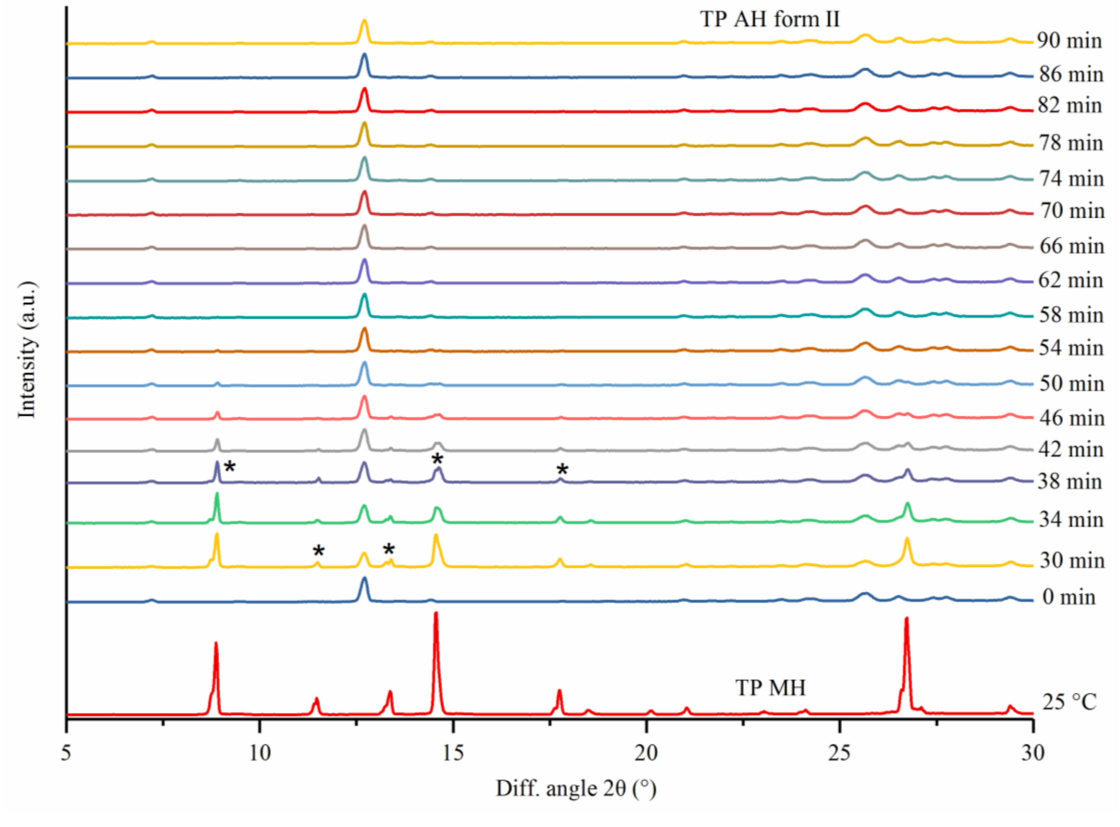

Supplementary figure 3. Variable temperature XRPD (VT-XRPD) of TP MH at 50 °C for 90 minutes. The diffractograms of TP MH is at 25 °C and TP AH form II at 90 minutes. The metastable intermediates appear to be present at 30 and 38 minutes into the experiment with the differences in peaks being marked by the asterix in comparison to their stable forms.

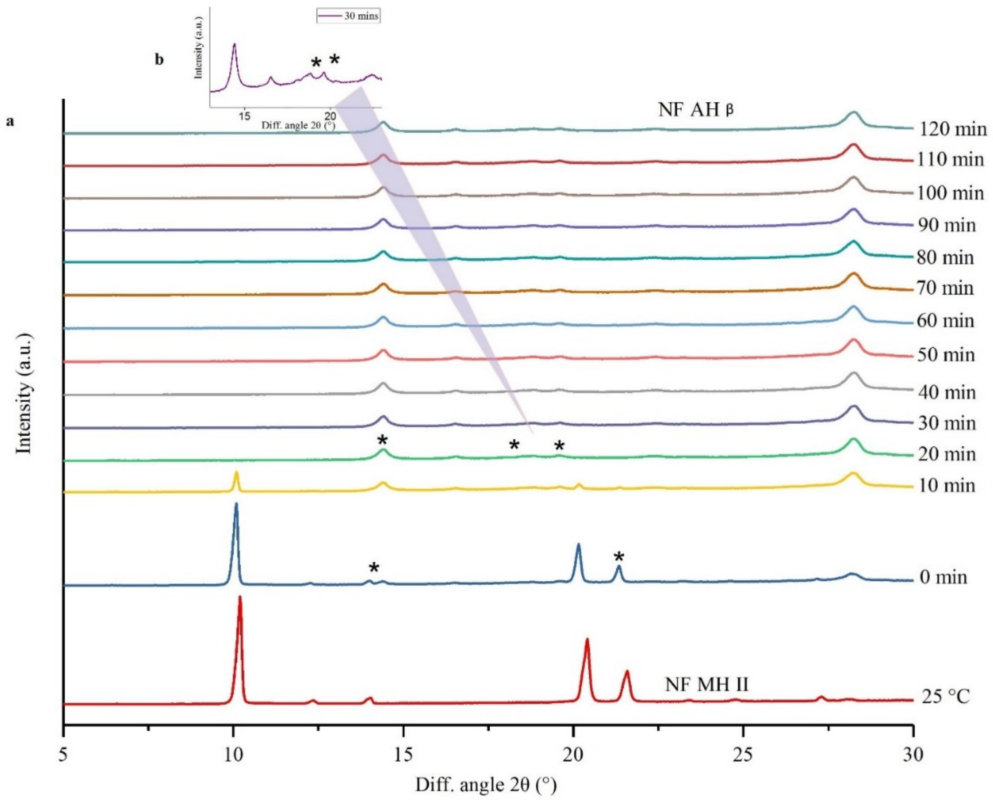

Supplementary figure 4. Variable temperature XRPD (VT-XRPD) of NF MH II at 120 °C for 120 minutes. The diffractogram of NF MH II is at 25 °C and NF AH β at 120 minutes. The metastable intermediates appear to be present at 0 and 30 minutes into the experiment with the differences in peaks being marked by the asterix in comparison to their stable forms. The zoom in of the peak features of potentially another intermediate are also shown at b.

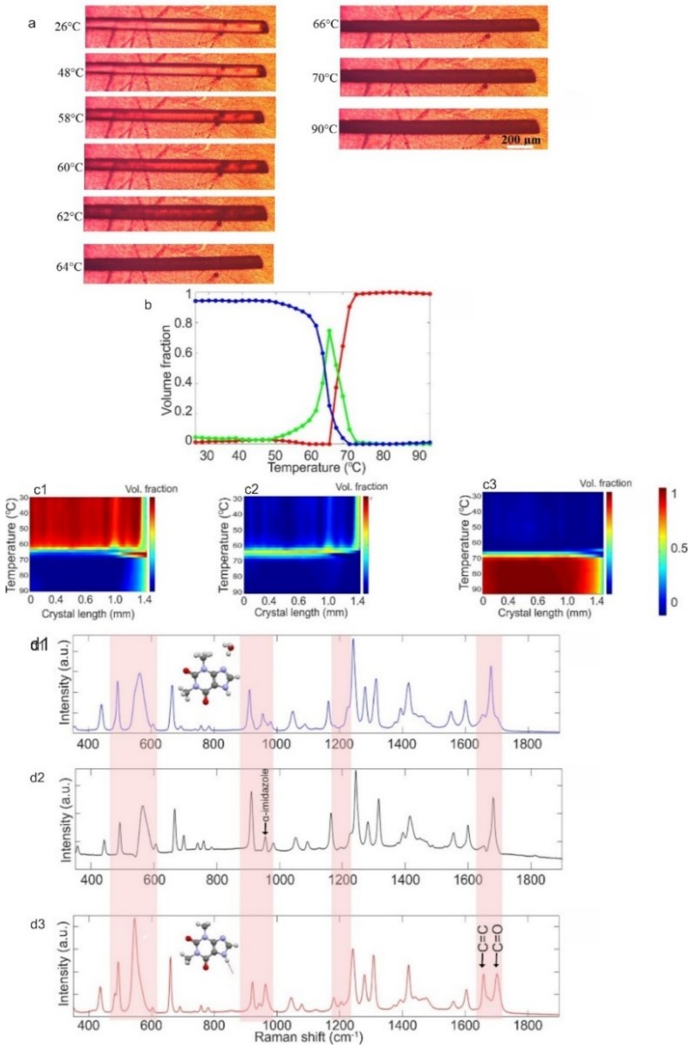

Supplementary figure 5: MCR and NNLS decomposed results of hydrated and dehydrated species of theophylline. a) Optical images showing the dehydration of TP MH to TP AH form II b) concentration profile of TP MH to TP AH form II **c1-c3)** chemical temperature dependent maps of TP MH (left map, red colour), TP MS (middle map, green colour) and TP AH form II (right map, blue colour) **d1-3)** Raman spectra for TP MH, TP MS and TP AH form II respectively.

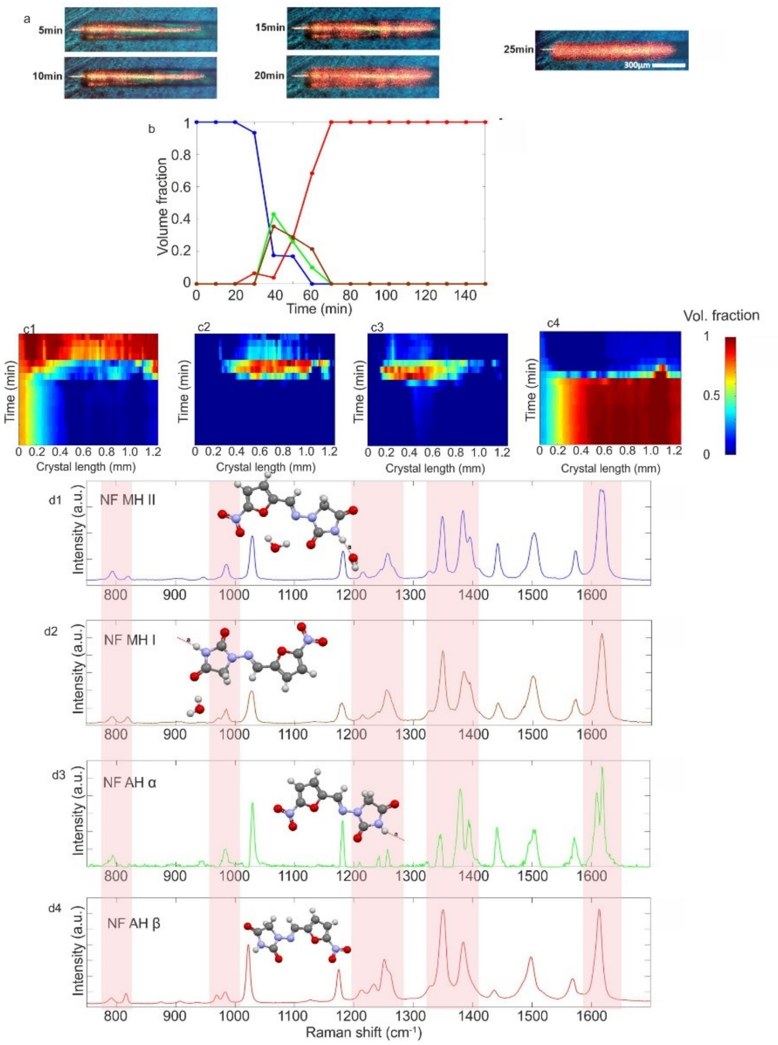

Supplementary figure 6: MCR and NNLS decomposed results of nitrofurantoin solid-state forms. a) Optical images showing dehydration of NF MH II to NF AH β. **b)** concentration profile of NF MH II dehydration mechanism to NF AH (β) **c1-c4)** chemical concentration maps and **d1-d4)** Raman spectra of NF MH II, NF MH I, NF AH α, NF AH β.

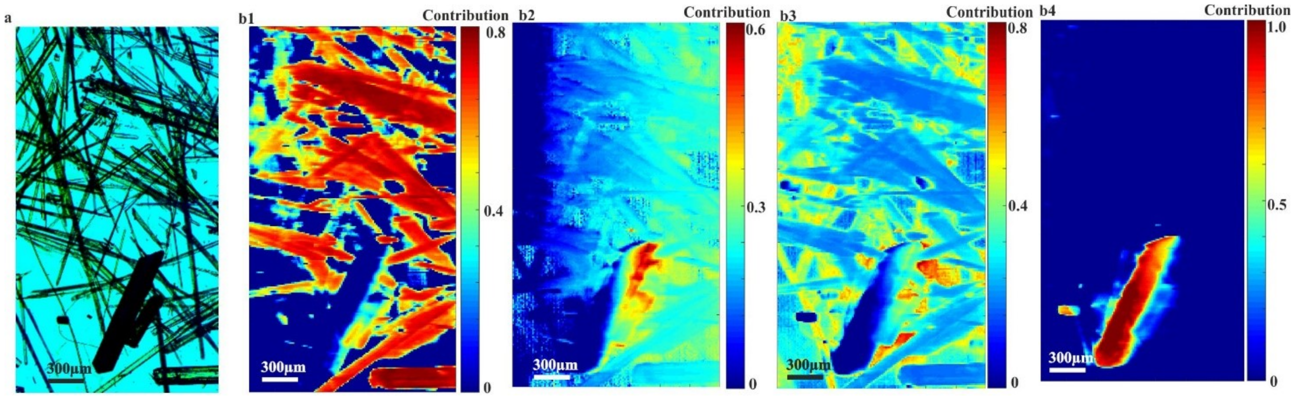

Supplementary figure 7. Multiple particles of NF solid-state forms analysis using the Raman line-focus method at room temperature (uncut) a) Optical images NF forms. **b1)** NF MH II **b2)** NF MH I **b3)** NF AH α and **b4)** NF AH β obtained after NNLS of a depolarised Raman map.
